# Integration of Proteomics and Metabolomics Revealed Metabolite–Protein Networks in ACTH-Secreting Pituitary Adenoma

**DOI:** 10.3389/fendo.2018.00678

**Published:** 2018-11-23

**Authors:** Jie Feng, Qi Zhang, Yang Zhou, Shenyuan Yu, Lichuan Hong, Sida Zhao, Jingjing Yang, Hong Wan, Guowang Xu, Yazhuo Zhang, Chuzhong Li

**Affiliations:** ^1^Beijing Neurosurgical Institute, Beijing Tiantan Hospital, Capital Medical University, Beijing, China; ^2^Beijing Institute for Brain Disorders, Brain Tumor Center, Capital Medical University, Beijing, China; ^3^China National Clinical Research Center for Neurological Diseases, Beijing Tiantan Hospital, Capital Medical University, Beijing, China; ^4^Department of Hepatobiliary and Pancreatic Surgery, The Second Affiliated Hospital, Zhejiang University School of Medicine, Hangzhou, China; ^5^CAS Key Laboratory of Separation Science for Analytical Chemistry, Dalian Institute of Chemical Physics, Chinese Academy of Sciences, Dalian, China

**Keywords:** metabolite–protein networks, proteomics, metabolomics, ACTH, pituitary adenoma

## Abstract

An effective treatment for the management of adrenocorticotropic hormone-secreting pituitary adenomas (ACTH-PA) is currently lacking, although surgery is a treatment option. We have integrated information obtained at the metabolomic and proteomic levels to identify critical networks and signaling pathways that may play important roles in the metabolic regulation of ACTH-PA and therefore hopefully represent potential therapeutic targets. Six ACTH-PAs and seven normal pituitary glands were investigated via gas chromatography-mass spectrometry (GC-MS) analysis for metabolomics. Five ACTH-PAs and five normal pituitary glands were subjected to proteomics analysis via nano liquid chromatography tandem-mass spectrometry (nanoLC-MS/MS). The joint pathway analysis and network analysis was performed using MetaboAnalyst 3.0. software. There were significant differences of metabolites and protein expression levels between the ACTH-PAs and normal pituitary glands. A proteomic analysis identified 417 differentially expressed proteins that were significantly enriched in the Myc signaling pathway. The protein–metabolite joint pathway analysis showed that differentially expressed proteins and metabolites were significantly enriched in glycolysis/gluconeogenesis, pyruvate metabolism, citrate cycle (TCA cycle), and the fatty acid metabolism pathway in ACTH-PA. The protein–metabolite molecular interaction network identified from the metabolomics and proteomics investigation resulted in four subnetworks. Ten nodes in subnetwork 1 were the most significantly enriched in cell amino acid metabolism and pyrimidine nucleotide metabolism. Additionally, the metabolite–gene–disease interaction network established nine subnetworks. Ninety-two nodes in subnetwork 1 were the most significantly enriched in carboxylic acid metabolism and organic acid metabolism. The present study clarified the pathway networks that function in ACTH-PA. Our results demonstrated the presence of downregulated glycolysis and fatty acid synthesis in this tumor type. We also revealed that the Myc signaling pathway significantly participated in the metabolic changes and tumorigenesis of ACTH-PA. This data may provide biomarkers for ACTH-PA diagnosis and monitoring, and could also lead to the development of novel strategies for treating pituitary adenomas.

## Introduction

Adrenocorticotropic hormone (ACTH)-secreting pituitary adenoma (ACTH-PA), also known as Cushing disease, is a monoclonal functioning pituitary adenoma that secretes excessive ACTH, which can cause multisystem symptoms, including central obesity, diabetes, hypertension, and psychiatric consequences. Although the majority of ACTH-PAs are benign, they are usually associated with high morbidity and mortality (1, 2). To date, tumor radiation and/or medical suppression of cortisol production have been used to treat this disease, but the efficacy is remains debatable. Surgery is the predominant treatment option, yet patients may suffer from recurrence. Unfortunately, an alternative treatment for the adequate management of ACTH-PA is currently lacking. A deeper understanding of the molecular mechanisms of ACTH-PA initiation and progression is warranted to develop novel strategies to treat this disease.

Tumor metabolic reprogramming has been considered a hallmark of cancer (3). Many oncogenes and suppressor genes play key roles in regulating the metabolism of tumor cells in order to support their growth and survival. These genes include but are not limited to, *Ras, Myc, HIF1A*, and *Tp53* (4). Tumors generally utilize glycolysis for energy production, which meet the requirements of both rapid growth and macromolecule biosynthesis. Many glycolytic enzymes are upregulated in tumors because of elevated c-Myc and HIF-1α transcriptional activities. In contrast, p53 is known to suppress glucose uptake by directly inhibiting the transcription of glucose transporters Glut1 and Glut4 and by suppressing the expression of Glut3 (4–6). An increase in lipid metabolism is another prominent feature of cancer metabolism. Lipid synthesis is a multistep process involving several enzymes, such as ATP citrate lyase (ACLY), fatty acid synthase (FASN), and stearoyl-CoA desaturase (SCD). FASN is a target gene of HIF-1α and is frequently upregulated by hypoxia (7). In addition, several studies have demonstrated that c-Myc promotes both glutamine uptake and the catabolic process of glutamine (4).The activity of glucose-6-phosphate dehydrogenase (G6PD), a critical enzyme participating in the pentose phosphate pathway, was reported to be increased in cancer cells. In fact, G6PD function is tightly controlled by p53. However, to date, the mechanism of abnormal metabolism in ACTH-PA is yet to be understood. Therefore, we have focused on ACTH-PA to investigate the metabolic and protein changes related to tumorigenesis.

Tumor is a complex disease and many high-throughput “-omic” technologies (genomics, transcriptomics, proteomics, and metabolomics) have been applied to tumors to study large-scale biological processes (BP) (8–10). The data generated from “-omic” studies have also driven the rapid development of integrative omics, whose aim is to integrate the information obtained from different levels of omic experiments into one unified model and to address the network of interactions and regulatory events that characterize the essential underlying biology.

In the present study, through gas chromatography-mass spectrometry (GC-MS) analysis and nano liquid chromatography tandem-mass spectrometry (nanoLC-MS/MS), we describe and integrate the data from the metabolomic and proteomic levels to identify critical networks and signaling pathways that may play important roles in the metabolic regulation of ACTH-PA, and therefore hope to elucidate potential therapeutic targets.

## Materials and methods

### Patients and specimens

All six patients without preoperative treatment suffered from hypercortisolemia with Cushing disease. The cortisol level of each patient is listed in Supplementary Table 1. The diagnosis of ACTH-PA was based on pathological and electron microscopic examination, as previously described. The low/high dose dexamethasone suppression tests supported the diagnosis. All six patients were diagnosed with functioning ACTH-PA and received trans-sphenoidal surgery at Beijing Tiantan Hospital. Fresh tumor tissue samples from these patients were frozen and stored in liquid nitrogen. Patients who had previously received radiation therapy or experienced tumor recurrence were not included in this study. All six functioning ACTH-PAs were used for metabolomic analysis, and five of them were used for proteomic analysis.

Seven healthy pituitary glands were used as controls. All control donors died from accidents and their pituitary glands had not been damaged. Written informed consent for the healthy donors was obtained from the next of kin. All seven pituitary glands were used for metabolomic analysis, and five of them were used for proteomic analysis.

This study was approved by the ethics committees of the Beijing Tiantan Hospital (KY2013-015-02). Informed consent was obtained from all of the enrolled subjects, and the study was performed in full compliance with all principles of the Helsinki Declaration.

### Protein preparation and NanoLC-MS/MS analysis

**Figure 1:**
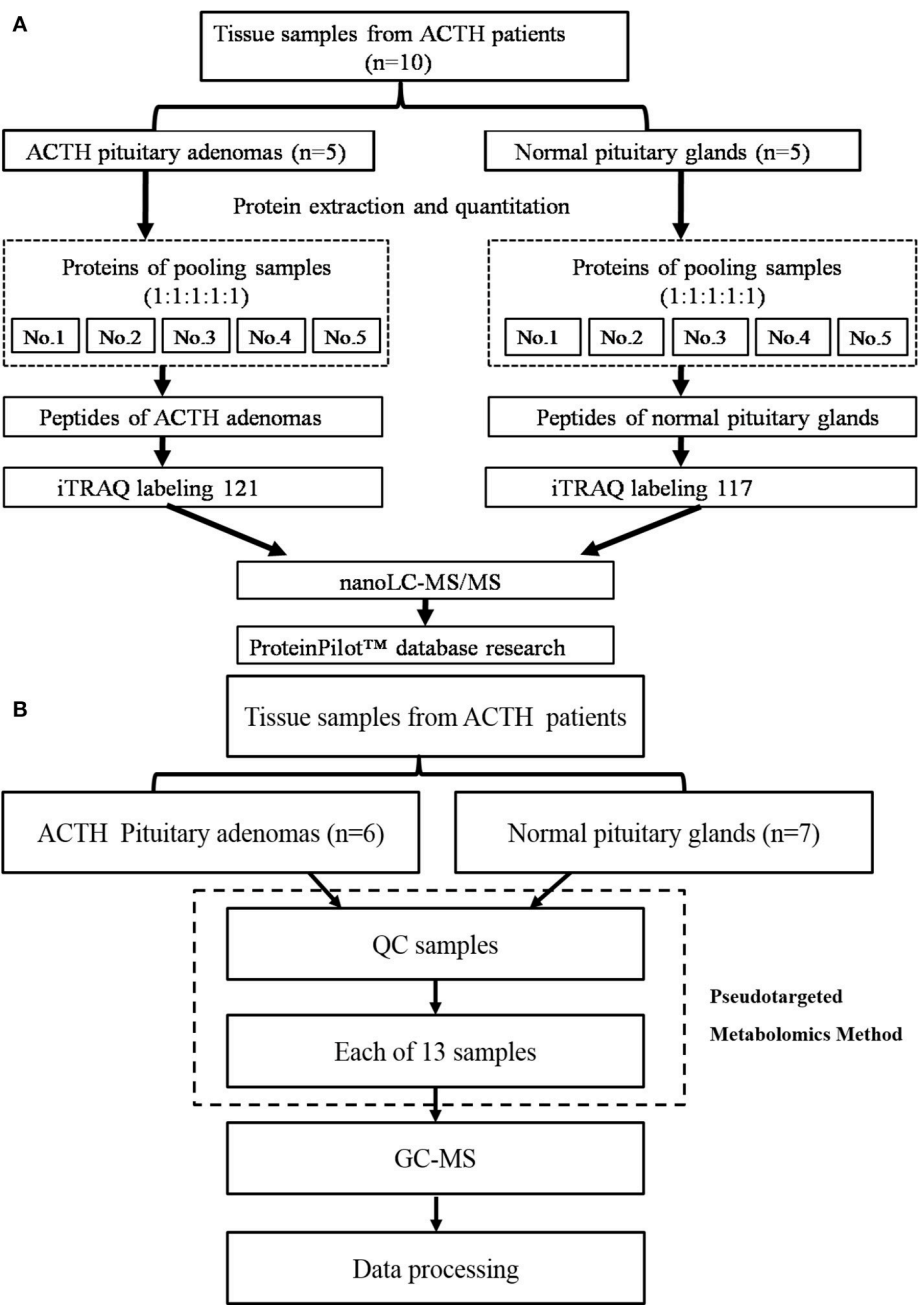
**(A)** The workflow of the protein preparation and nano LC-MS/MS analysis, **(B)** The workflow of the metabolomics analysis.

The workflow of the protein preparation and nanoLC-MS/MS analysis are shown in Figure 1A. The proteins were extracted using a total protein extraction kit (2140, Millipore, Billerica, MA, USA). The protein concentrations were measured using a bicinchoninic acid protein assay kit (23225, Pierce, Rockford, IL, USA).

The proteins from five ACTH-PAs or five healthy pituitary glands were equally combined into a single pool, as previously described (11). The pooling of samples in proteomics should reduce the measured biological variation giving increased power to detect treatment differences (12, 13). A total of 100 μg of each pooled sample was denatured, reduced, and alkylated, as described in the iTRAQ protocol (Applied Biosystems Sciex, USA) and digested overnight with 0.1 μg/μL trypsin solution at 37°C. The digested ACTH-PA and healthy pituitary gland pooled samples were labeled with 121 and 117 iTRAQ tags, respectively, according to the manufacturer's protocol (Applied Biosystems Sciex, USA). The tagged peptides were dried via vacuum centrifugation and combined in one tube. Strong cation-exchange (SCX) chromatography was performed according to a previously described method (11). Briefly, the pooled sample was separated on an apoly-LC SCX column (4.6 × 250 mm, 5 μm, 100 Å) using an LC 100 instrument (Eksigent, Dublin, CA, USA), and the labeled peptides were detected by ultraviolet radiation using an SPD-20 (Shimadzu, Japan). In this study, a total of 48 fractions were collected, dried by speed vacuum centrifugation, and combined into 10 fractions according to the SCX chromatogram. Each fraction was injected onto a desalting column (350 μm × 0.5 mm, 3 μm C18, 120 Å) and separated on an analytical column (75 μm × 150 mm, 3 μm C18, 120 Å) using an Eksigent nanoLC instrument (Eksigent, Dublin, CA, USA). The samples were separated via capillary high-performance liquid chromatography and were subsequently analyzed using a Triple TOF 5600 system (Applied Biosystems Sciex, USA).

Protein identification and differentially expression were performed using the ProteinPilot software package (Applied Biosystems Sciex, USA) and searched against the SwissProt database (March 2013) using the Mascot 2.2 search engine (Matrix Science, London, UK). The following search parameters were utilized to analyze the MS/MS data: trypsin as the digestion enzyme, with a maximum of two missed cleavages allowed; fixed modifications of carbamidomethyl (C) and iTRAQ Plex (K and N-terminus); variable modifications of oxidation (M); peptide mass tolerance of ±20 ppm; fragment mass tolerance of ± 0.1 Da; and peptide FDR ≤ 0.01.

### Metabolomics and GC-MS analysis

The workflow of metabolomics analysis is shown in Figure 1B. For GC-MS analysis, tissue samples were mixed with 600 μl of a methanol/water (v/v 4:1) solution containing internal standards and homogenate. Supernatants were lyophilized for subsequent oximation and silylation reactions. A QP 2010 GC-MS system (Shimadzu, Japan) with a DB-5 MS fused-silica capillary column (30 m × 0.25 mm × 0.25 μm, Agilent Technologies, Santa Clara, CA, USA) was used for metabolic profiling. A pseudotargeted GC-MS metabolomics method was established elsewhere (14–16). The ion peak area of the metabolite was normalized to the internal standard and multiplied by 1 × 10^6^, then utilized for following data processing. A total of 288 features assigned to 32 groups were defined for data collection and quantification. The system parameter settings have previously been described (16). Metabolite identities were determined based on commercial libraries (Mainlib, NIST, Wiley, and Fiehn) and an internal metabolite library.

### Bioinformatic analysis and statistics

Student's *t*-test and SAM were performed to calculate the differential expression and false discovery rate (FDR) between ACTH-PAs and normal pituitary glands. Filtering was performed to identify metabolites that were either overexpressed or underexpressed by at least 2.0-fold and to determine *q-*values of < 5% in ACTH-PAs compared with normal pituitary glands. Comprehensive metabolomic data analysis was performed by using MetaboAnalyst 3.0 (http://www.metaboanalyst.ca/faces/home.xhtml).

Hierarchical cluster analysis was performed to create a heatmap of the differentially expressed metabolites using MetaboAnalyst 3.0. Protein enrichment pathway analysis was used based on the hallmark gene set database determined by Gene Set Enrichment Analysis software (GSEA, http://software.broadinstitute.org/gsea/msigdb/index.jsp). The joint pathway analysis conducted using MetaboAnalyst 3.0 enabled the visualization of significant genes and metabolites that were enriched in a particular pathway. Network analysis was performed by MetaboAnalyst 3.0 in three different modes: gene-metabolite interaction network, metabolite–disease interaction network, and metabolite–gene–disease interaction network. Functional annotation databases were utilized based on the BP determined by gene ontology (GO).

## Results

### Hierarchical clustering of metabolic profiling in ACTH-PA

**Figure 2:**
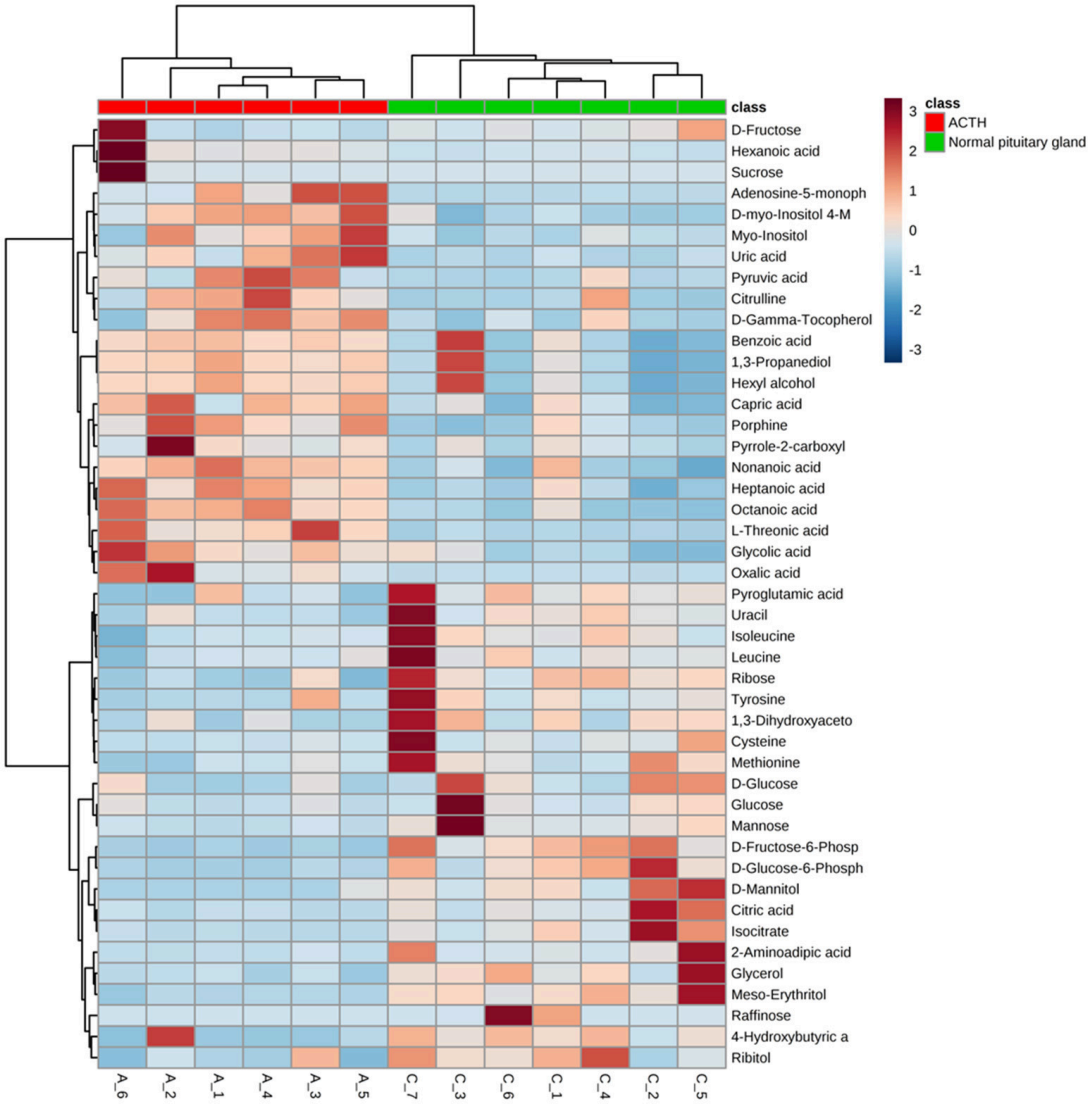
A heatmap illustrating that the 37 metabolites clearly segregate patients with ACTH-PAs and normal pituitary glands. Each colored cell on the map corresponds to a concentration value in the data table, with samples in rows and features/compounds in columns. The heatmap was used to identify samples/features that are unusually high/low.

Significant differences of metabolites between ACTH-PAs (*n* = 6) and normal pituitary glands (*n* = 7) were observed. A total of 192 metabolites were identified among the ACTH-PAs and normal pituitary glands, and 37 of these metabolites were diversely expressed between the two groups (*P-*value < 0.05, with a fold change >2 or < 0.5). Specifically, 17 metabolites were upregulated and 20 were downregulated in ACTH-PA samples. A heatmap with two-dimensional hierarchical clustering (Figure 2) illustrated that the analyzed metabolites clearly segregated the samples into two groups and was consistent with the clinical diagnosis of the patients.

### Protein enrichment pathway analysis in ACTH-PA

We next explored the proteins that were differentially expressed between ACTH adenomas (*n* = 5) and normal pituitary glands (*n* = 5). A proteomic analysis identified 48,391 peptides that were mapped to 4,568 proteins in this study. A total of 417 differentially expressed proteins were further identified (*P* < 0.05; FDR < 0.01; iTRAQ ratio >2 or < 0.5). Of these proteins, 218 and 199 were upregulated and downregulated in ACTH-PAs, respectively, compared to the normal pituitary glands.

**Figure 3:**
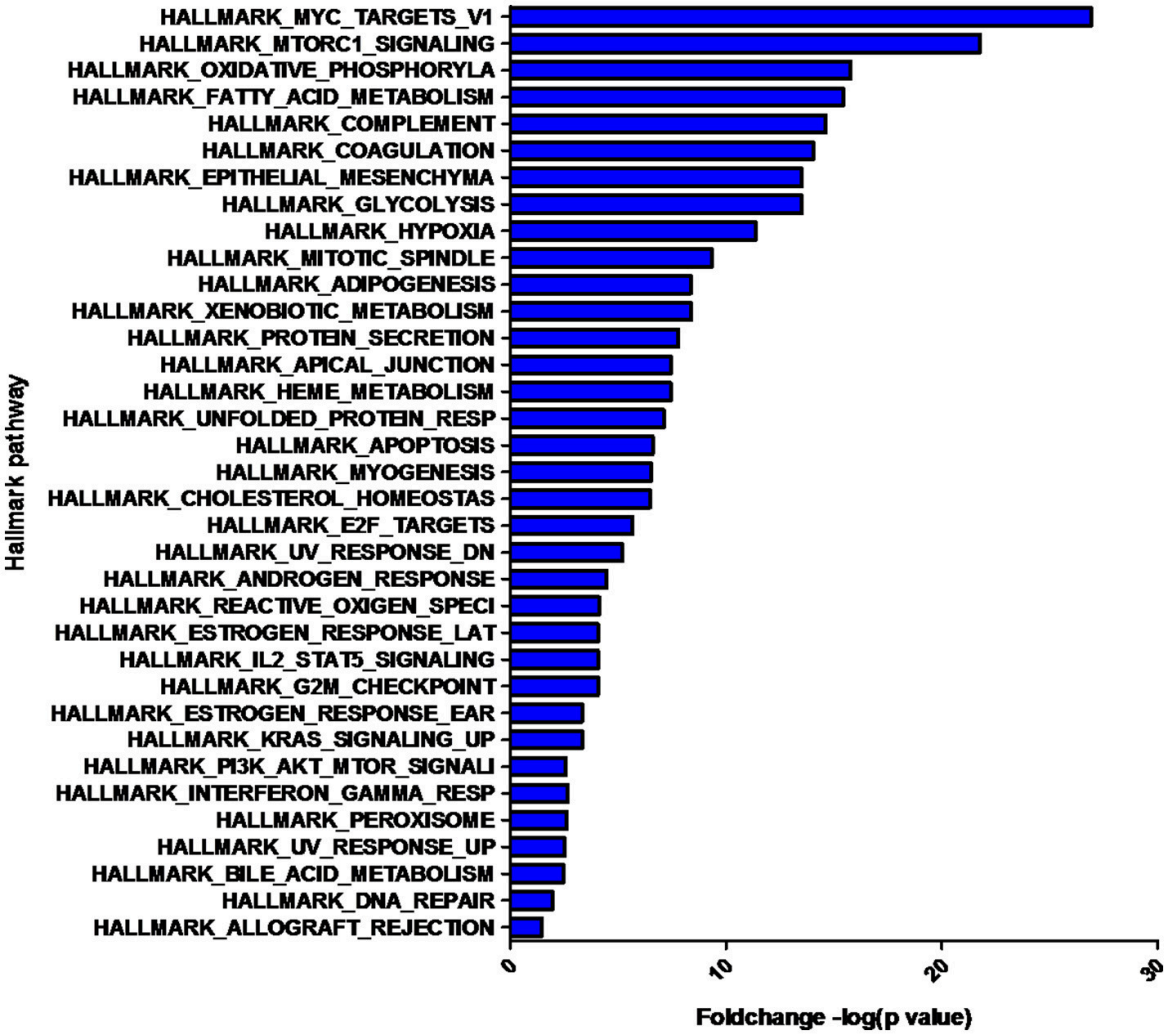
Hallmark pathways enriched by the differentially expressed proteins. The significant pathways are displayed along the X-axis. The Y-axis displays the –log of the *p*-value.

**Figure 4:**
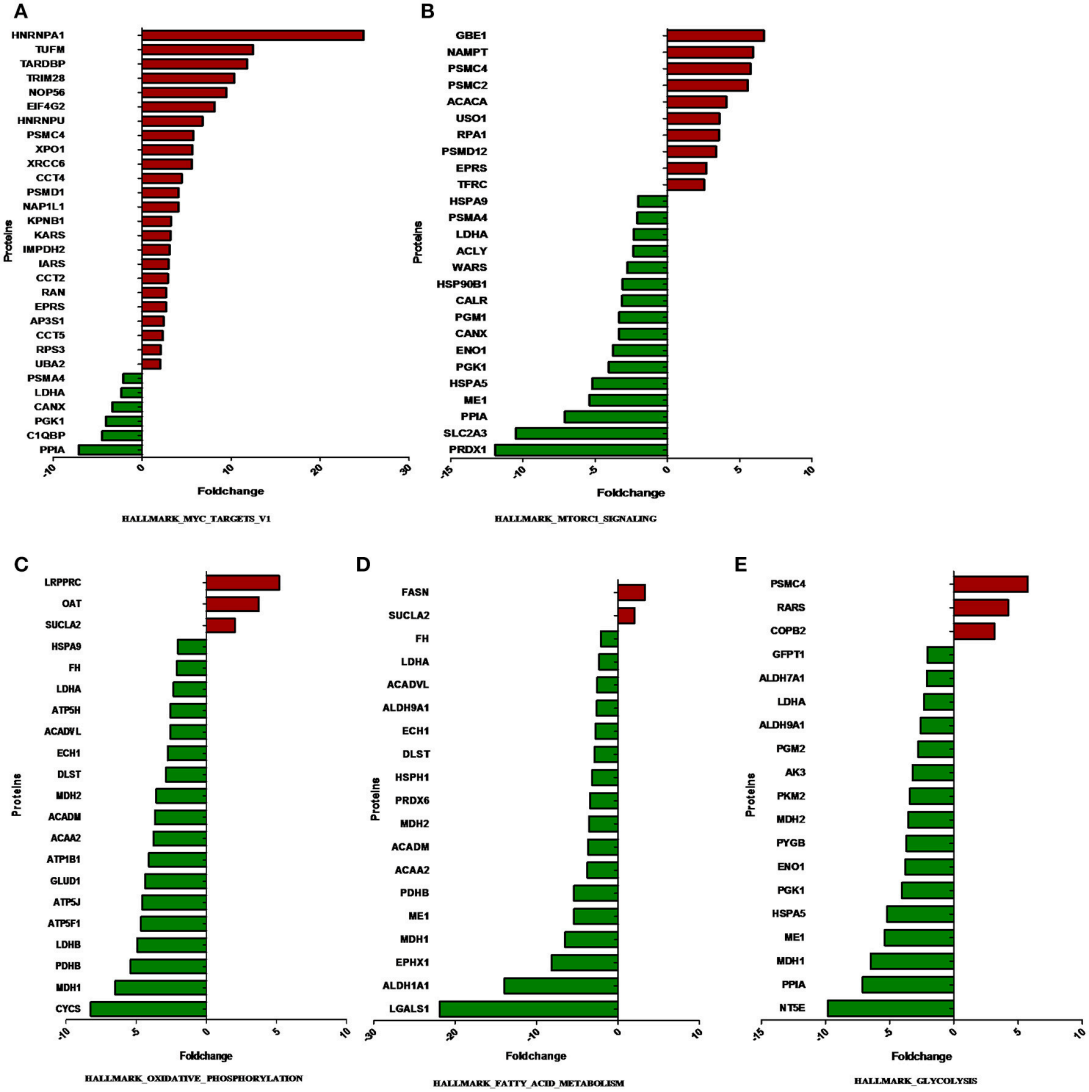
The expression of proteins in metabolism-related hallmark pathways. The proteins differentially expressed between ACTH-PAs and normal pituitary glands are displayed along the X-axis. The Y-axis displays the –log of the *p*-value. **(A)** Proteins in HALLMARK_MYC_TARGETS_V1, **(B)** Proteins in HALLMARK_MTORC1_SIGNALING, **(C)** Proteins in HALLMARK_OXIDATIVE_PHOSPHORYLATION, **(D)** Proteins in HALLMARK_FATTY_ACID_METABOLISM, **(E)** Proteins in HALLMARK_GLYCOLYSIS.

The overlap computing tool of GSEA evaluates the overlap of a provided differentially expression protein/gene set with hallmark gene sets from MSigDB and estimates the statistical significance. The protein/gene sets with a *P-*value < 0.05 and an FDR *q-*value < 0.05 are shown in Figure 3. These hallmark pathways were closely related to tumor metabolism and included HALLMARK_MYC_TARGETS_V1, HALLMARK_MTORC1_SIGNALING, HALLMARK_OXIDATIVE_PHOSPHORYLATION, HALLMARK_FATTY_ACID_METABOLISM, and HALLMARK_GLYCOLYSIS. The expression of proteins in metabolism-related hallmark pathways is listed (Figure 3). Intriguingly, the majority of proteins in the HALLMARK_OXIDATIVE_PHOSPHORYLATION, HALLMARK_FATTY_ACID_METABOLISM, and HALLMARK_GLYCOLYSIS pathways were found to be downregulated (Figures 4C–E).

### Protein–metabolite joint pathway analysis

**Figure 5:**
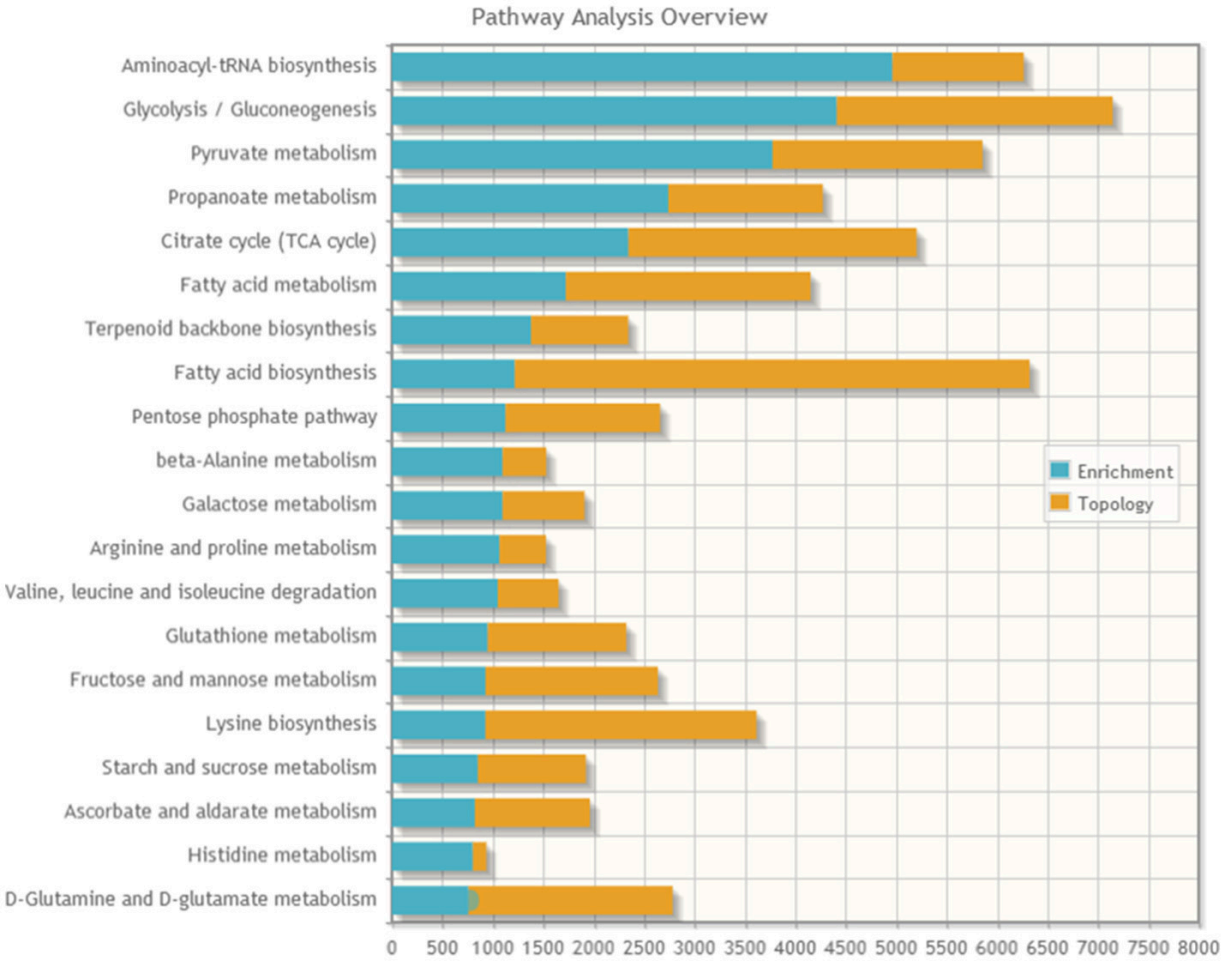
The stacked bars below show a summary of the protein–metabolite joint evidence from enrichment analysis and topology analysis.

A joint pathway analysis was performed using the enrichment analysis and the topology analysis. The enrichment analysis showed the identified proteins and metabolites that were significantly enriched in a particular pathway (*P* < 0.05; Figure 5), including aminoacyl-tRNAbiosynthesis, glycolysis/gluconeogenesis, pyruvate metabolism, propanoate metabolism, citrate cycle (TCA cycle), fatty acid metabolism, terpenoid backbone biosynthesis, fatty acid biosynthesis, pentose phosphate pathway, beta-alanine metabolism, and galactose metabolism. The topology analysis showed the identified genes or metabolites that probably play an important role in pathways based on their positions within these pathways.

### Protein–metabolite interaction network

**Figure 6:**
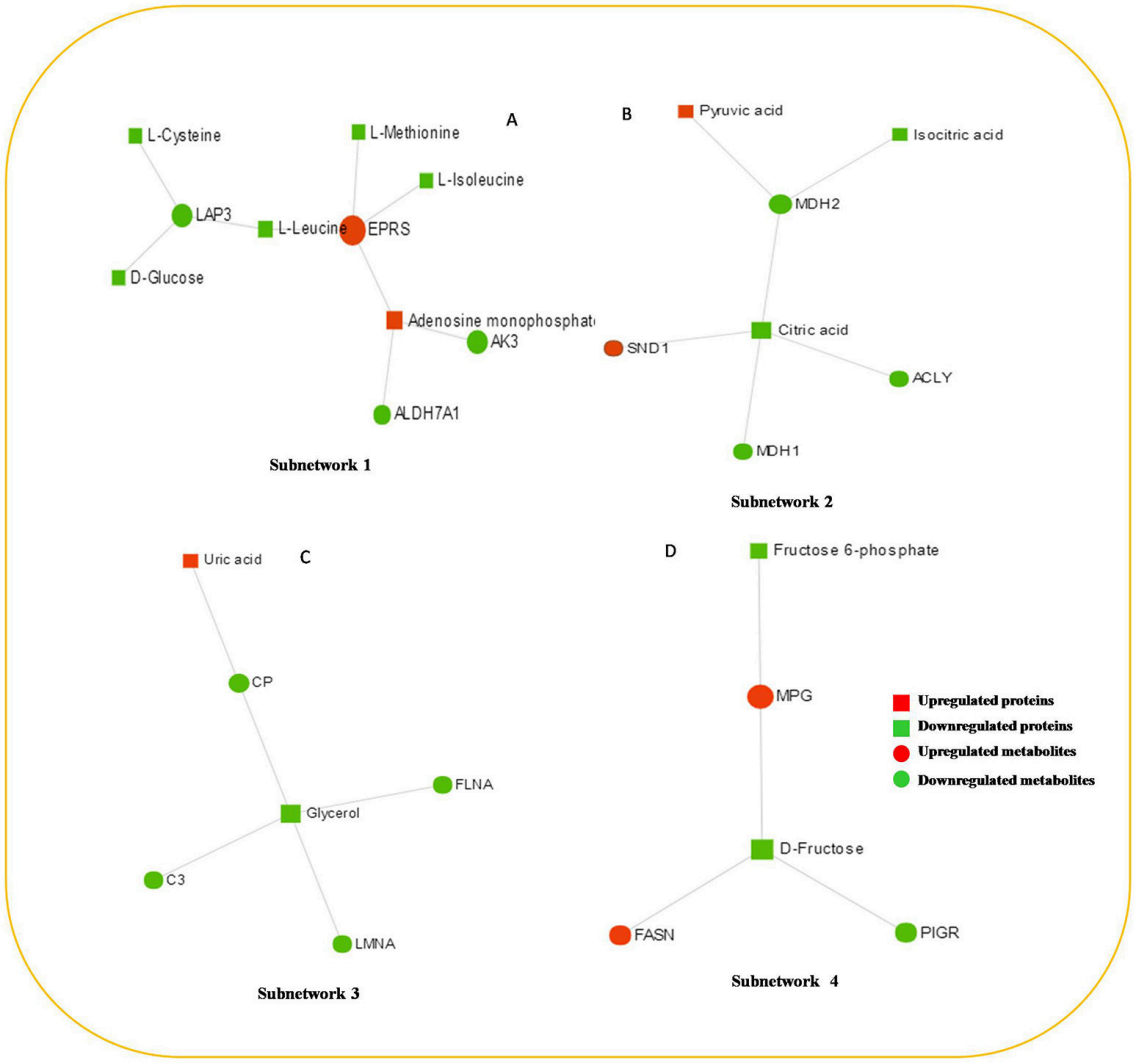
The protein–metabolite interaction network provides a visualization of the interactions between functionally related metabolites and genes (proteins) identified from proteomics and metabolomics. **(A)** Subnetwork 1, **(B)** Subnetwork 2, **(C)** Subnetwork 3, **(D)** Subnetwork 4.

The protein–metabolite interaction network provides visible interactions between functionally related metabolites and proteins. The metabolites and proteins identified from proteomics and metabolomics were mapped to the protein–metabolite molecular interaction network to create four subnetworks (Figure 6).

Subnetwork 1 includes 10 nodes (proteins, metabolites), and two of them were upregulated, including EPRS and adenosine monophosphate (AMP) (Figure 6A). The nodes (proteins, metabolites) in subnetwork 1 were the most significantly enriched in cell amino acid metabolism and pyrimidine nucleotide metabolism based on the GO:BP database (Table 1).

**Table 1:** Pathways enriched by proteins and metabolites in subnetwork 1 of the protein–metabolite interaction network based on the GO:BP database.

**Pathway**	**Total**	**Expected**	**Hits**	***P*-value**
Cellular amino acid metabolic process	670	0.188	2	0.0124
Pyrimidine nucleotide metabolic process	50	0.014	1	0.0139
Negative regulation of translation	70	0.0196	1	0.0195
Cellular modified amino acid biosynthetic process	71	0.0199	1	0.0197
Carboxylic acid metabolic process	1,270	0.357	2	0.0422
Cellular amino acid catabolic process	166	0.0465	1	0.0457
Cellular biogenic amine metabolic process	167	0.0468	1	0.0459
tRNA metabolic process	173	0.0484	1	0.0476
Organic acid metabolic process	1,430	0.4	2	0.0522

Subnetwork 2 includes seven nodes (proteins, metabolites), and two of them were upregulated, including SND1 and pyruvate acid (Figure 6B). The nodes in subnetwork 2 were the most significantly enriched in cellular carbohydrate metabolism and aerobic respiration based on the GO:BP database (Table 2).

**Table 2:** Pathways enriched by proteins and metabolites in subnetwork 2 of the protein–metabolite interaction network based on the GO:BP database.

**Pathway**	**Total**	**Expected**	**Hits**	***P*-value**
Cellular carbohydrate metabolic process	259	0.0725	3	2.32*E*−05
Aerobic respiration	61	0.0171	2	0.000107
Energy derivation by oxidation of organic compounds	437	0.122	3	0.000111
Generation of precursor metabolites and energy	603	0.169	3	0.00029
Carbohydrate biosynthetic process	203	0.0568	2	0.00118
Carbohydrate metabolic process	1,040	0.291	3	0.00145
Cellular respiration	236	0.0661	2	0.00159
Coenzyme metabolic process	266	0.0745	2	0.00202
Glucose metabolic process	290	0.0812	2	0.0024
Carboxylic acid metabolic process	1,270	0.357	3	0.00264
Cofactor metabolic process	331	0.0927	2	0.00311
Organic acid metabolic process	1,430	0.4	3	0.00369
Gene silencing	99	0.0277	1	0.0274
Nucleotide metabolic process	1,040	0.292	2	0.0289
Triglyceride metabolic process	126	0.0353	1	0.0348
Coenzyme biosynthetic process	133	0.0372	1	0.0367

Subnetwork 3 includes six nodes (proteins, metabolites), and only uric acid was upregulated (Figure 6C). The nodes in subnetwork 3 were the most significantly enriched in protein import into the nucleus and nuclear import based on the GO:BP database (Table 3).

**Table 3:** Pathways enriched by proteins and metabolites in subnetwork 3 of the protein–metabolite interaction network based on the GO:BP database.

**Pathway**	**Total**	**Expected**	**Hits**	***P*-value**
Protein import into nucleus	228	0.0638	2	0.00149
Nuclear import	232	0.0649	2	0.00154
Protein import	272	0.0761	2	0.00211
Microtubule cytoskeleton organization	337	0.0943	2	0.00323
Nucleocytoplasmic transport	388	0.109	2	0.00426
Nuclear transport	392	0.11	2	0.00434
Cellular membrane organization	471	0.132	2	0.00623
Microtubule-based process	516	0.144	2	0.00744
Regulation of protein metabolic process	1,820	0.511	3	0.00753
Protein targeting	545	0.153	2	0.00828

Subnetwork 4 includes five nodes (proteins, metabolites), two of which were upregulated, including MPG and FASN (Figure 6D). The nodes in subnetwork 4 were the most significantly enriched in base-excision and the DNA catabolic process based on the GO:BP database (Table 4).

**Table 4:** Pathways enriched by proteins and metabolites in subnetwork 4 of the protein–metabolite interaction network based on the GO:BP database.

**Pathway**	**Total**	**Expected**	**Hits**	***P*-value**
Base-excision repair	45	0.0063	1	0.00629
DNA catabolic process	72	0.0101	1	0.0101
DNA modification	83	0.0116	1	0.0116
Vitamin metabolic process	115	0.0161	1	0.016
Triglyceride metabolic process	126	0.0176	1	0.0176
Coenzyme biosynthetic process	133	0.0186	1	0.0185
Fatty acid biosynthetic process	151	0.0211	1	0.021
Cofactor biosynthetic process	185	0.0259	1	0.0257
Energy reserve metabolic process	199	0.0279	1	0.0277
Cellular modified amino acid metabolic process	241	0.0337	1	0.0335
Coenzyme metabolic process	266	0.0372	1	0.0369
Cofactor metabolic process	331	0.0463	1	0.0458

### Metabolite–disease interaction network

**Figure 7:**
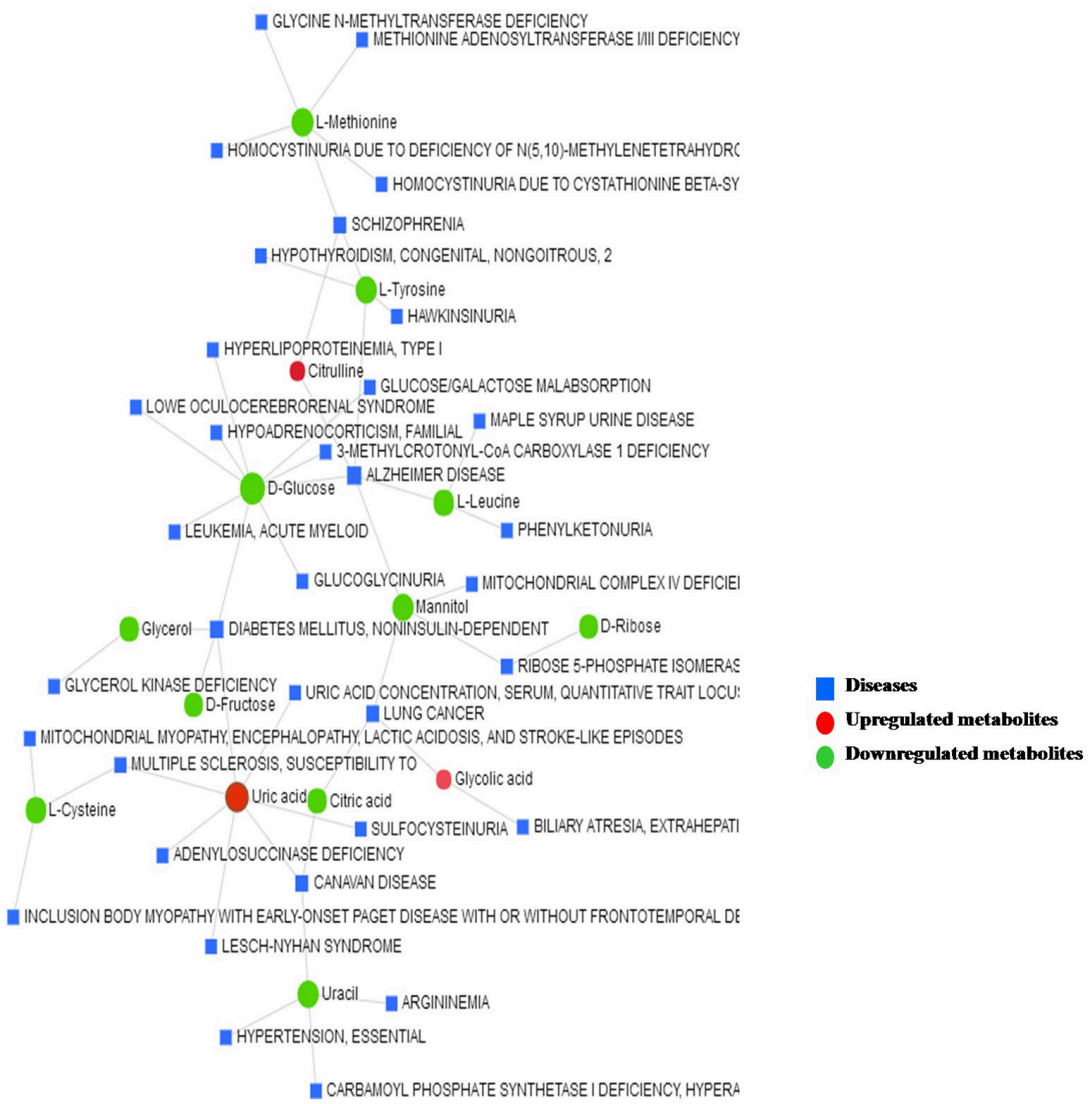
The metabolite–disease interaction network provides a visualization of the disease-related metabolites identified from metabolomics.

The metabolite–disease interaction network provides an exploration of disease-related metabolites. The metabolites identified from metabolomics were mapped to the metabolite–disease interaction network to create two subnetworks (Figure 7).

Subnetwork 1 includes 48 nodes (metabolites, connected proteins, and target diseases), and 14 of them were metabolites identified from the present study. In detail, 3 of the 14 metabolites were upregulated: uric acid, citrulline, and glycolic acid (Figure 7).

### Metabolite–protein–disease interaction network

The metabolite–protein–disease interaction network provides a global view of potentially functional relationships between metabolites, connected proteins, and target diseases. The metabolites and proteins identified from the metabolomics and proteomics analyses were mapped to the metabolite–gene–disease interaction network and successfully created nine subnetworks.

**Figure 8:**
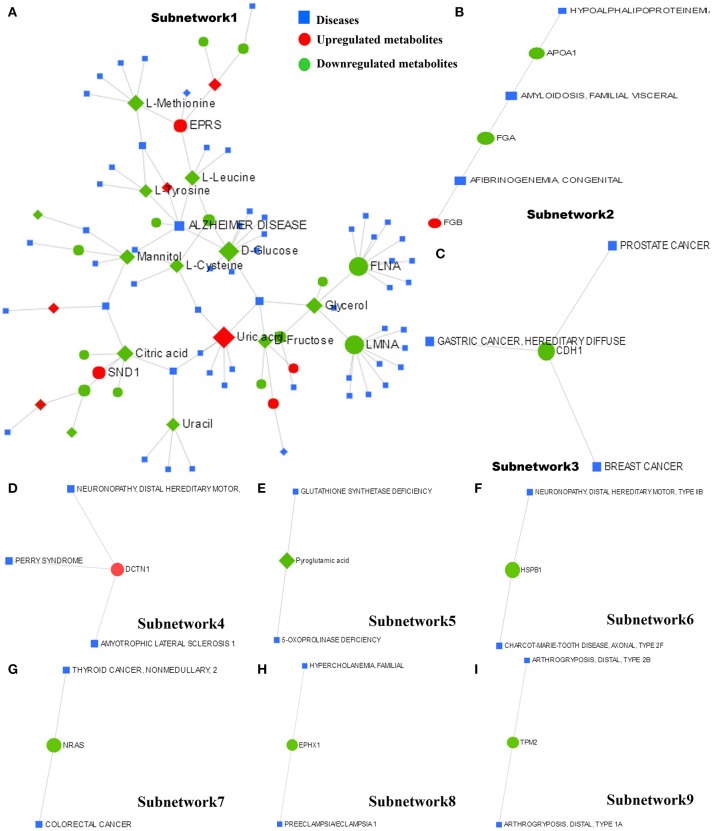
The metabolite–protein–disease interaction network provides a global view of the potential functional relationships between metabolites, connected proteins identified from proteomics and metabolomics and target diseases. The nine subnetworks are indicated in **(A–I)**, respectively.

Subnetwork 1 includes 92 nodes (metabolites, connected proteins, and target diseases), and 36 of them were metabolites and proteins identified from the present study. Five of the 36 metabolites and proteins were upregulated: uric acid, EPRS, AMP, glycolic acid, and FASN (Figure 8A). Figure 8A indicates the potentially functional relationships between metabolites such as D-glucose, L-cysteine, L-tyrosine, L-leucine, mannitol, and Alzheimer's disease.

Subnetwork 2 includes six nodes, and three of them were proteins identified from the present study. FGB was indicated to be upregulated (Figure 8B). Subnetwork 2 showed potential functional relationships between proteins FGB, FGA, APOA1, and afibrinogenemia congenital, amyloidosis familial visceral, and hypoalphalipoproteinemia.

The other 7 subnetworks (3–9) are also shown in Figure 8. Subnetwork 3 and subnetwork 4 include four nodes, and one node consisted of proteins identified from the current study. CHD1 in subnetwork 3 was indicated to have a potentially functional relationship with tumors such as breast cancer, gastric cancer, and prostate cancer. DCTN1 in subnetwork 4 was shown to have a potentially functional relationship with neuronopathy distal hereditary motor, Perry syndrome, and amyotrophic lateral sclerosis 1.

Subnetworks 5–9 include three nodes, one of which was a protein or metabolite identified from our study. The metabolite pyroglutamic acid in subnetwork 5 has a potentially functional relationship with diseases such as glutathione synthetase deficiency and 5-oxoprolinase deficiency. NRAS in subnetwork 7 was indicated to have a potentially functional relationship with colorectal cancer and thyroid cancer. HSPB1 in subnetwork 6, EPHX1 in subnetwork 8, and TPM2 in subnetwork 9 were indicated to have potentially functional relationships with diseases such as neuronopathy distal hereditary motor, Charcot-Marie-Tooth disease axonal, hypercholanemia familial, preeclampsia/eclampsia 1, and arthrogryposis distal.

## Discussion

The present study, for the first time, used integrative omic analysis from proteomics and metabolomics to reveal the significant molecular signaling pathways and networks that have potentially functional relationship with ACTH-PA. Among the complicated pathway networks described above, several protein–metabolite joint pathways and networks were found to be significantly associated with the abnormal metabolism in ACTH-PA.

### Glycolysis/gluconeogenesis

It is well-known that tumor cells preferentially use glycolysis for their energy supply. The majority of glycolytic enzymes were markedly elevated in most tumors. In addition to their metabolic functions, glycolytic enzymes also play important roles in cell survival, metastasis, invasion, chromatin remodeling, regulation of gene expression, and other essential cellular processes (4, 17). In addition, glycolysis provides cancer cells not only with energy but also with the necessary precursors for biosynthesis. For example, several glycolytic metabolites, such as glucose-6-phosphate and pyruvate, could be diverted into other metabolic pathways. Furthermore, lactate not only is taken up by other cancer cells in the tumor microenvironment to enhance TCA flux but also lowers the pH of the extracellular microenvironment, facilitating the activity of metalloproteases for tumor invasion (4, 18).

Notably, most of the proteins involved in glycolysis and glucogenesis, including LDHA, HK1, and PKM2, showed lower levels in ACTH-PAs than in normal pituitary glands (Figure 4). Some metabolites, such as glucose-6-phosphate, which is used for the synthesis of nucleotides and NADPH, were reduced in ACTH-PAs (Supplementary Tables 2, 3). These phenomena are markedly different from the Warburg effect seen in most tumors.

Furthermore, pyruvate was found to be significantly higher in ACTH-PAs compared to normal pituitary glands, but it appeared to not be diverted into mitochondrial TCA and lactate metabolism. We speculate that pyruvate was detoured into alanine metabolism.

### Fatty acid metabolism

An increase in fatty acid metabolism is another remarkable feature of tumor metabolism. Tumor cells upregulate fatty acid synthesis to meet their requirements for fatty acid. Fatty acid synthesis is a multistep process involving several critical enzymes. Fatty acid synthase (FASN) was reported to be elevated in many cancers, including breast, prostate and other types of cancer. However, fatty acid catabolic metabolism, including fatty acid oxidation, still remains a poorly understood metabolic pathway.

In the present study, FASN was shown to be upregulated in ACTH-PAs, which was similar to other types of cancer (4, 19), while enzymes related to fatty acid catabolic metabolism, such as ACADVL, ACADM, and ACAA2, were downregulated in ACTH-PAs (Figure 4). A limited number of metabolites from fatty acid metabolism were identified due to the GC-MS methods. Additionally, short chain fatty acids, such as capric acid, hexanoic acid, heptanoic acid, nonanoic acid, and octanoic acid, were found to be increased in ACTH-PAs (Table 5).

**Table 5:** The differentially expressed level of metabolites between ACTH-PAs and normal pituitary glands.

**Name**	***P*-value**	**Fold change**
Capric acid	0.010	1.65
Heptanoic acid	0.007	2.20
Hexanoic acid	0.003	2.26
Nonanoic acid	0.015	1.72
Octanoic acid	0.003	2.05
D-Glucose-6-phosphate	0.003	0.14

### Mitochondrial metabolism

Another major change in cancer metabolism is the abnormal mitochondrial biogenesis and metabolism. Mitochondria play essential roles in cancer cells because mitochondria are not only involved in energy production but also in the synthesis of many indispensable molecules for cellular biosynthesis, growth, and proliferation (20). In contrast to normal cells that use mitochondrial TCA and oxidative phosphorylation for energy production, tumor cells preferentially use glycolysis for energy production (4), which was similar to our results. Our data also showed that the proteins and metabolites involved in mitochondrial TCA and oxidative phosphorylation, such as MDH1, MDH2, FH, CYCS, ATP5H, ATP5J, ATP5F1, ATP1B1, citrate, and isocitrate, were reduced in ACTH-PAs (Figures 4, 5). Alternatively, mitochondrial biogenesis was increased in tumors. TUFM is a Tu translation elongation factor that participates in protein translation in mitochondria. In the present study, the expression of TUFM, which is indispensable for mitochondrial biogenesis, was shown to be increased in ACTH-PAs. Therefore, our results suggested enhanced mitochondrial biogenesis in ACTH-PA.

### Myc signaling pathway and metabolism

Our proteomic profiling results further indicated that Myc signaling was deeply involved in the altered metabolism of ACTH-PA. In the previous study, Myc as the master inducer of tumor glycolysis, promoted the expression of key glycolytic enzymes (21). Most glycolytic gene promoter areas contain consensus Myc-binding motifs. However, the expression of glycolytic enzymes such as LDHA and PGK1 in the Myc signaling pathway was decreased in ACTH-PA, which was consistent with the reduced glycolysis observed in this tumor type. It is thus possible that the Myc signaling pathway takes part in regulating the glycolysis of ACTH-PAs.

In addition, Myc upregulates glutaminolysis in tumor cells. Many studies have demonstrated that Myc promotes both glutamine uptake and the catabolic process of glutamine. Myc also participates in mitochondrial biogenesis and metabolism (22). This is associated with the transcriptional induction of TFAM, the proteins of the complex I subunits, uncoupling proteins, mitochondrial membrane proteins, and the proteins involved in intermediary metabolism (20). Although we did not find any proteins of the Myc signaling pathway related to glutaminolysis in ACTH-PA, we did notice that some proteins in the Myc signaling pathway that were associated with mitochondrial biogenesis (such as TUFM) were increased in ACTH-PA.

It has been reported that the Myc signaling pathway is often activated during tumorigenesis. Consistently, our proteomic profiling revealed that Myc signaling pathway proteins that are involved in protein and nucleotide synthesis, such as PSMC2, PSMC4, EIF4G2, and IMPDH2, had increased expression in ACTH-PA (Figure 4).

## Conclusions

The present study clarified pathway networks that function in ACTH-PA. Our results demonstrated a downregulated glycolysis and fatty acid synthesis in ACTH-PA. We also revealed that the Myc signaling pathway significantly participates in the metabolic changes and tumorigenesis of ACTH-PAs. Further experimental investigations are required to elucidate the biological consequences of these pathway networks and their relevance to the tumorigenesis of ACTH-PAs. The data from the current study may provide biomarkers for ACTH-PA diagnosis and monitoring, and possibly lead to the development of novel strategies to treat the tumors.

## Author contributions

CL and JF conceived the idea. CL and YazZ collected the samples. JF, SY, JY, and LH performed the proteomic analyses. YanZ and GX performed the metabolomic analysis. JF, HW, CL, and YazZ interpreted the data. QZ and JF aided in the data analysis and wrote the manuscript. All authors approved the submission.

### Conflict of interest statement

The authors declare that the research was conducted in the absence of any commercial or financial relationships that could be construed as a potential conflict of interest.
